# Myocardial CKIP-1 Overexpression Protects from Simulated Microgravity-Induced Cardiac Remodeling

**DOI:** 10.3389/fphys.2018.00040

**Published:** 2018-01-25

**Authors:** Shukuan Ling, Yuheng Li, Guohui Zhong, Yongjun Zheng, Qing Xu, Dingsheng Zhao, Weijia Sun, Xiaoyan Jin, Hongxing Li, Jianwei Li, Huiyuan Sun, Dengchao Cao, Jinping Song, Caizhi Liu, Xinxin Yuan, Xiaorui Wu, Yinlong Zhao, Zizhong Liu, Qi Li, Yingxian Li

**Affiliations:** ^1^State Key Lab of Space Medicine Fundamentals and Application, China Astronaut Research and Training Center, Beijing, China; ^2^Medical Administration Division, The 261th Hospital of PLA, Beijing, China; ^3^Core Facility Center, Capital Medical University, Beijing, China; ^4^Key Laboratory of Molecular and Cellular Biology of Ministry of Education, College of Life Science, Hebei Normal University, Shijiazhuang, China; ^5^Xiyuan Hospital, China Academy of Chinese Medical Sciences, Beijing, China; ^6^State Key Laboratory of Agrobiotechnology, College of Life Sciences, China Agricultural University, Beijing, China

**Keywords:** CKIP-1, simulated microgravity, cardiac remodeling, HDAC4, AMPK, ERK1/2

## Abstract

Human cardiovascular system has adapted to Earth's gravity of 1G. The microgravity during space flight can induce cardiac remodeling and decline of cardiac function. At present, the mechanism of cardiac remodeling induced by microgravity remains to be disclosed. Casein kinase-2 interacting protein-1 (CKIP-1) is an important inhibitor of pressure-overload induced cardiac remodeling by decreasing the phosphorylation level of HDAC4. However, the role of CKIP-1 in the cardiac remodeling induced by microgravity is unknown. The purpose of this study was to determine whether CKIP-1 was also involved in the regulation of cardiac remodeling induced by microgravity. We first detected the expression of CKIP-1 in the heart from mice and monkey after simulated microgravity using Q-PCR and western blotting. Then, myocardial specific CKIP-1 transgenic (TG) and wild type mice were hindlimb-suspended (HU) to simulate microgravity effect. We estimated the cardiac remodeling in morphology and function by histological analysis and echocardiography. Finally, we detected the phosphorylation of AMPK, ERK1/2, and HDAC4 in the heart from wild type and CKIP-1 transgenic mice after HU. The results revealed the reduced expression of CKIP-1 in the heart both from mice and monkey after simulated microgravity. Myocardial CKIP-1 overexpression protected from simulated microgravity-induced decline of cardiac function and loss of left ventricular mass. Histological analysis demonstrated CKIP-1 TG inhibited the decreases in the size of individual cardiomyocytes of mice after hindlimb unloading. CKIP-1 TG can inhibit the activation of HDAC4 and ERK1/2 and the inactivation of AMPK in heart of mice induced by simulated microgravity. These results demonstrated CKIP-1 was a suppressor of cardiac remodeling induced by simulated microgravity.

## Introduction

Human cardiovascular system has adapted to Earth's gravity of 1G, and cardiac muscle is well regulated in response to changes in loading conditions (Tuday and Berkowitz, 2007; Hill and Olson, 2008). When exposed to microgravity during space flight, there are various changes in cardiac mass and cardiac systolic volume (Hill and Olson, 2008). Microgravity can induce the chronic reduction in metabolic demand and oxygen uptake which reduces the demand on cardiac output, resulting in cardiac atrophy and the decline of cardiac function (Dorfman et al., 2007; Zhong G. et al., 2016; Zhong G. H. et al., 2016). There are many important factors which can regulate cardiac remodeling induced by external or intrinsic stimuli, including AMPK, ERK1/2, and HDAC4 (Ling et al., 2012; Ruppert et al., 2013; Myers et al., 2017). Our previous study demonstrated that pathological cardiac remodeling signals, such as HDAC4 and ERK1/2, were activated, and physiological cardiac remodeling signals, such as AMPK, were inactivated in heart of mice after hindlimb unloading, which might lead to cardiac remodeling and decline of heart function (Zhong G. et al., 2016). Beyond that, we know little about the mechanisms regulating cardiac atrophy induced by microgravity. Also, the prevention of cardiac remodeling induced by simulated microgravity via inhibiting the changes of these signals activity need to be elucidated.

The PH domain-containing casein kinase 2 interacting protein-1 (CKIP-1) was first identified as an interactive protein of casein kinase 2a (Bosc et al., 2000), and functions as a scaffold protein integrating multiple signaling pathways and is highly expressed in the heart under physiological functions (Ling et al., 2012). CKIP-1 was implicated in tumor cell proliferation (Zhu et al., 2017), muscle cell differentiation (Safi et al., 2004), cell apoptosis (Zhang et al., 2005), the regulation of cell morphology (Canton et al., 2006), and the actin cytoskeleton (Canton et al., 2005). We previously showed that CKIP-1 functioned as a novel regulator of pathological cardiac remodeling induced by pressure overload (Ling et al., 2012). We found that CKIP-1-deficent mice displayed age-dependent cardiac hypertrophy and decline of cardiac function. Pressure overload-induced cardiac hypertrophy was exaggerated in CKIP-1-deficient mice. Moreover, myocardial-specific CKIP-1 overexpression protects from cardiac hypertrophy induced by pressure overload. The anti-hypertrophic effects of CKIP-1 are mediated by dephosphorylation and nuclear retention of HDAC4. CKIP-1 is a potential target for inhibiting cardiac remodeling induced by pressure overload (Ling et al., 2012). As with other form of cardiac- remodeling cardiac hypertrophy induced by pressure overload, cardiac atrophy induced by microgravity although linked with different phenotypes (atrophy vs. hypertrophy), generate interesting similar changes-upregulation of cardiac remodeling marker genes and decline of cardiac function (Depre et al., 1998). However, the role of CKIP-1 in cardiac remodeling induced by microgravity was unknown.

In this study, we found CKIP-1 mRNA and protein levels in hearts of mice and rhesus monkeys after simulated microgravity were significantly decreased. The results showed myocardial CKIP-1 overexpression protected from the decline of cardiac function, the atrophy of cardiomyocyte and changes in phosphorylation levels of signal factors induced by simulated microgravity. We demonstrated myocardial CKIP-1 overexpression protected from cardiac remodeling induced by simulated microgravity.

## Materials and methods

### Animal experiments

All animal studies were performed according to approved guidelines for the use and care of live animals (Guideline on Administration of Laboratory Animals released in1988 and 2006 Guideline on Humane Treatment of Laboratory Animals from China, and also referring to European Union guideline 2010/63). The experimental procedures were approved by the Animal Care and Use Committee of China Astronaut Research and Training Center.

The healthy rhesus monkeys with body weight of 5–8 kg and 4–8 years old were purchased from Beijing Xieerxin Biology Resource (Beijing, China). The monkeys were maintained−10-degree head-down tilt position for 42 days. The whole process were supervised and monitored 24 h/day. This 42-day bed rest experiment of rhesus monkeys have been reported previously (Chen et al., 2016).

Myocardial-specific CKIP-1 transgenic mice have been reported previously (Ling et al., 2012). All WT and CKIP-1 transgenic (TG) mice used in this study were bred and housed at the specific-pathogen-free (SPF) Animal Research Center of China Astronaut Research and Training Center (12:12-h light-dark cycle, temperature: 23°C). All the experiments were repeated three times performed with CKIP-1 mice (2 month) and age-matched WT controls. The hindlimbs of hindlimb-unloading procedure were elevated by tail suspension, as described before (Zhong G. et al., 2016). Briefly, the 2 months old mice were maintained in individual cage and suspended with a strip attached the tail and linked a chain hanging from a pulley. The mice were elevated to an angle of 30° to the ground, and only the forelimbs of mice can touch the floor, so the hindlimb-unloading mice can move freely to eat and drink. The mice were retained to hindlimb unloading by tail suspension for 28 days, the height of hindlimb suspension was modulated to prevent the hinklimb from touching the ground. This is identified as the “unloaded” state. Similar numbers of control mice of the age-matched littermates and the same strain background were instrumented and monitored in the identical cage conditions without tail suspension.

### Histological analysis

Sections were generated from paraffin embedded hearts, and were stained with H&E for gross morphology, Masson's trichrome for detection of fibrosis, Frozen sections were used to visualize cardiomyocyte cell membranes by staining with TRITC-conjugated wheat-germ agglutinin (Sigma-Aldrich), as described before (Ling et al., 2012).

### RNA extraction and real-time polymerase chain reaction

Total RNA was extracted from heart tissues by using RNAiso Plus reagent (Takara) according to the manufacturer's protocol. The RNA was reverse transcribed into cDNA, and qPCR was performed using a SYBR Green PCR kit (Takara) in a Light Cycler (LightCycler 96, Roche, USA). The mRNA level of each gene was normalized to that of Gapdh, which served as an internal control. Primers (synthesized by Sunbiotech Co, China) for *Col1a1, Col3a1*, BNP, *Ckip1*, and *Gapdh* were as follows:

Mouse:

*Col1a1* sense primer: 5′-CTGACTGGAAGAGCGGAGAGT-3′,
*Col1a1* anti-sense primer: 5′-AGACGGCTGAGTAGGGAACAC-3′;
*Col3a1* sense primer: 5′-ACGTAAGCACTGGTGGACAG-3′,
*Col3a1* anti-sense primer: 5′-CAGGAGGGCCATAGCTGAAC-3′;
BNP sense primer: 5′-TGTTTCTGCTTTTCCTTTATCTG-3′,
BNP anti-sense primer: 5′-TCTTTTTGGGTGTTCTTTTGTGA-3′;
*CKIP-1* sense primer: 5′-GCCGTGAGTCCTGAAGAGAAG-3′;
*CKIP-1* anti-sense primer: 5′-CGAGTAGGGTGGGCAAGATAG-3′;
*Gapdh* sense primer: 5′-ACTCCACTCACGGCAAATTCA-3′;
*Gapdh* anti-sense primer: 5′-GGCCTCACCCCATTTGATG-3′.

Monkey:

*Col1a1* sense primer: 5′-TGACGAGACCAAGAACTGCC-3′,
*Col1a1* anti-sense primer: 5′-CAGGAGATTACCTCGACGCC-3′;
*Col3a1* sense primer: 5′-CAAAAGGGGAGCTGGCTACT-3′,
*Col3a1* anti-sense primer: 5′-CAACAGCTTCCTGTTGTGCC-3′;
BNP sense primer: 5′-AATGGTCCTGTACACCCTGC-3′,
BNP anti-sense primer: 5′-ATCTTCCTCCCAAAGCAGCC-3′;
*CKIP-1* sense primer: 5′-TCAGGATGGAAACCAGCA-3′;
*CKIP-1* anti-sense primer: 5′-TTCAGCACCACATAGCGGT-3′;
*Gapdh* sense primer: 5′-CGAGAGTCAGCCGCATTTTC-3′;
*Gapdh* anti-sense primer: 5′-GACTCCGACCTTCACCTTCC-3′.

### Echocardiography

Animals were lightly anesthetized with 2,2,2-tribromoethanol (0.2 ml/10 g body weight of a 1.2% solution) and set in a supine position. Two dimensional (2D) guided M-mode echocardiography was performed using a high resolution imaging system (Vevo 2100, Visual-Sonics Inc., Toronto, ON, Canada). Two-dimensional images are recorded in parasternal long- and short-axis projections with guided M-mode recordings at the midventricular level in both views. Left ventricular (LV) cavity size and wall thickness are measured in at least three beats from each projection. Averaged LV wall thickness [anterior wall (AW) and posterior wall (PW) thickness] and internal dimensions at diastole and systole (LVIDd and LVIDs, respectively) are measured. LV fractional shortening ((LVIDd–LVIDs)/LVIDd), relative wall thickness ((IVS thickness + PW thickness)/LVIDd), and LV mass (LV Mass = 1.053 × [(LVID;d + LVPW;d + IVS;d)3 – LVID;d3) are calculated from the M-mode measurements. LV ejection fraction (EF) wascalculated from the LV cross-sectional area (2-D short-axis view) using the equation LV%EF = (LV Vol;d – LV Vol;s)/LV Vol;d × 100%. The studies and analysis were performed blinded as to experimental groups. Structure and function of LV were assessed as described before (Ling et al., 2012).

### Western blot analysis

Hearts of mouse and monkey were crushed by homogenizer (Power Gen125, Fisher Scientific) and then lysed in lysis buffer (50 mM Tris, pH 7.5, 250 mM NaCl, 0.1% SDS, 2 mM dithiothreitol, 0.5% NP-40, 1 mM PMSF and protease inhibitor cocktail) on ice for 15 min. Protein fractions were collected by centrifugation for 15 min (12,000 g, 4°C) and then applied to 10% SDS-PAGE gels, electrophoresed at 80 V for 30 min and 120 V for 90 min. After electrophoresis, protein was transfected to a polyvinylidene fluoride membrane using a Criterion blotter apparatus (Bio Rad). The membrane was then blocked in 5% non-fat dry milk (Becton, Dickinson and Company) in TBST (10 mM Tris–Cl, 150 mM NaCl, 0.05% Tween-20, pH 7.5) for 2 h. After that, the membrane was incubated with primary antibody overnight at 4°Cfollowed by incubation with a secondary antibody conjugated to horseradish peroxidase (HRP), and visualized using an chemiluminescence kit (Thermo Pierce, No.32 109). Specific antibodies to p-HDAC4 (Cell Signaling Technology, #3443S), HDAC4 (Cell Signaling Technology, #5392S), p-AMPKα (Cell Signaling Technology, #2531S), AMPKα (Cell Signaling Technology, #2532), p-ERK1/2 (Cell Signaling Technology, #4370S), ERK1/2 (Cell Signaling Technology, #4696S), CKIP-1 (proteintech, #24883-1), Gapdh (Santa Cruz Biotechnology, sc-25778) were used to detect protein levels. Gapdh was used as a loading control.

### Statistical analysis

Data are presented as mean ± SEM per experimental condition. Statistical differences among groups were analyzed by one-way analysis of variance with a *post-hoc* test to determine group differences in the study parameters. Statistical differences between two groups were determined by the Student's *t*-test. We use the two-way analysis of variance with unequal variances to account for 2 factors and their interactions. Firstly, we test the equality of variances using a factorial effects analysis of variance on the absolute values of the residuals. If the variances are unequal, we then fit a mixed model with heterogeneous variances. Otherwise, we use the regular linear model. Bonferroni adjustment was used for multiple comparisons. *P* < 0.05 is considered statistically significant. All the statistical tests are analyzed by Prism software (Graphpad prism for windows, version 5.01) and SPSS (Version 14.0).

## Results

### The changes of CKIP-1 expression in the hearts of mice and rhesus monkeys after simulated microgravity

**Figure 1:**
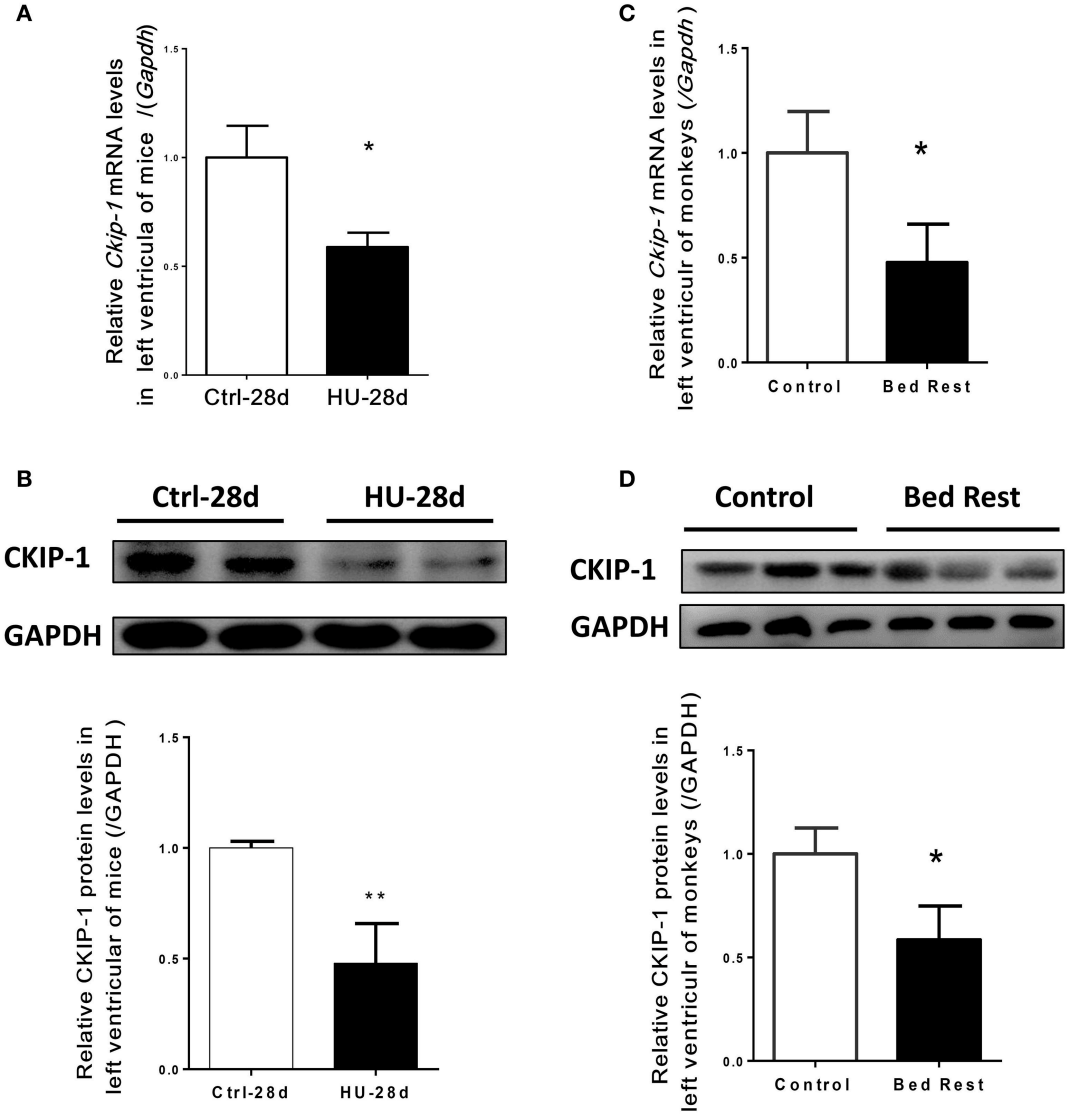
CKIP-1 expression changes in in the hearts of mice and rhesus monkeys after simulated microgravity. **(A)** CKIP-1 mRNA levels change in left ventricle of mice after 28 days hindlimb unloading (*n* = 6). **(B)** Representative Western blotting analysis of CKIP-1 expression in cardiac extracts of adult mice after 28 days hindlimb unloading. **(C)** CKIP-1 mRNA levels change in left ventricle of rhesus monkeys following 45 days of head-down bed rest (*n* = 3). **(D)** Western blotting analysis of CKIP-1 expression in cardiac extracts of rhesus monkey after 45 days bed rest. ^**^P < 0.01, ^*^P < 0.05.

To assess the potential role of CKIP-1 in cardiac remodeling induced by simulated microgravity, hearts from mice after 28 days of hindlimb unloading were assessed for CKIP-1 expression. As shown in Figures 1A,B, CKIP-1 mRNA and protein levels were significantly decreased in the hearts of mice after hindlimb unloading. Simulated microgravity can downregulate CKIP-1 expression in mice heart. Moreover, we detected CKIP-1 expression in hearts of rhesus monkeys after 45 days of bed rest. Firstly, real-time polymerase chain reaction analysis showed that the transcripts of the pathological cardiac remodeling genes-Col1a1, Col3a1, BNP, and β-MHC were constantly increased in the hearts of monkey after bed rest, however no obvious changes were showed in α-MHC mRNA level (Supplementary Figure 1). And the changes in phosphorylation levels of HDAC4, AMPK, and ERK1/2 which were proved to be involved in cardiac remodeling of mice after hind limb unloading were detected in hearts of rhesus monkey after 45 days of bed rest. The results showed that following 45 days of bed rest, pathological cardiac remodeling signals-HDAC4 and ERK1/2 were activated, and physiological cardiac remodeling signal-AMPK were inactivated in monkey hearts (Supplementary Figure 2). Meantime, CKIP-1 mRNA and protein levels were also significantly decreased in the hearts of rhesus monkeys after bed rest (Figures 1C,D). These results indicated that simulated microgravity reduced the mRNA and protein levels of CKIP-1 which was a suppressor of pathological cardiac remodeling.

### Myocardial CKIP-1 overexpression protects from simulated microgravity induced-decline of cardiac function

**Figure 2:**
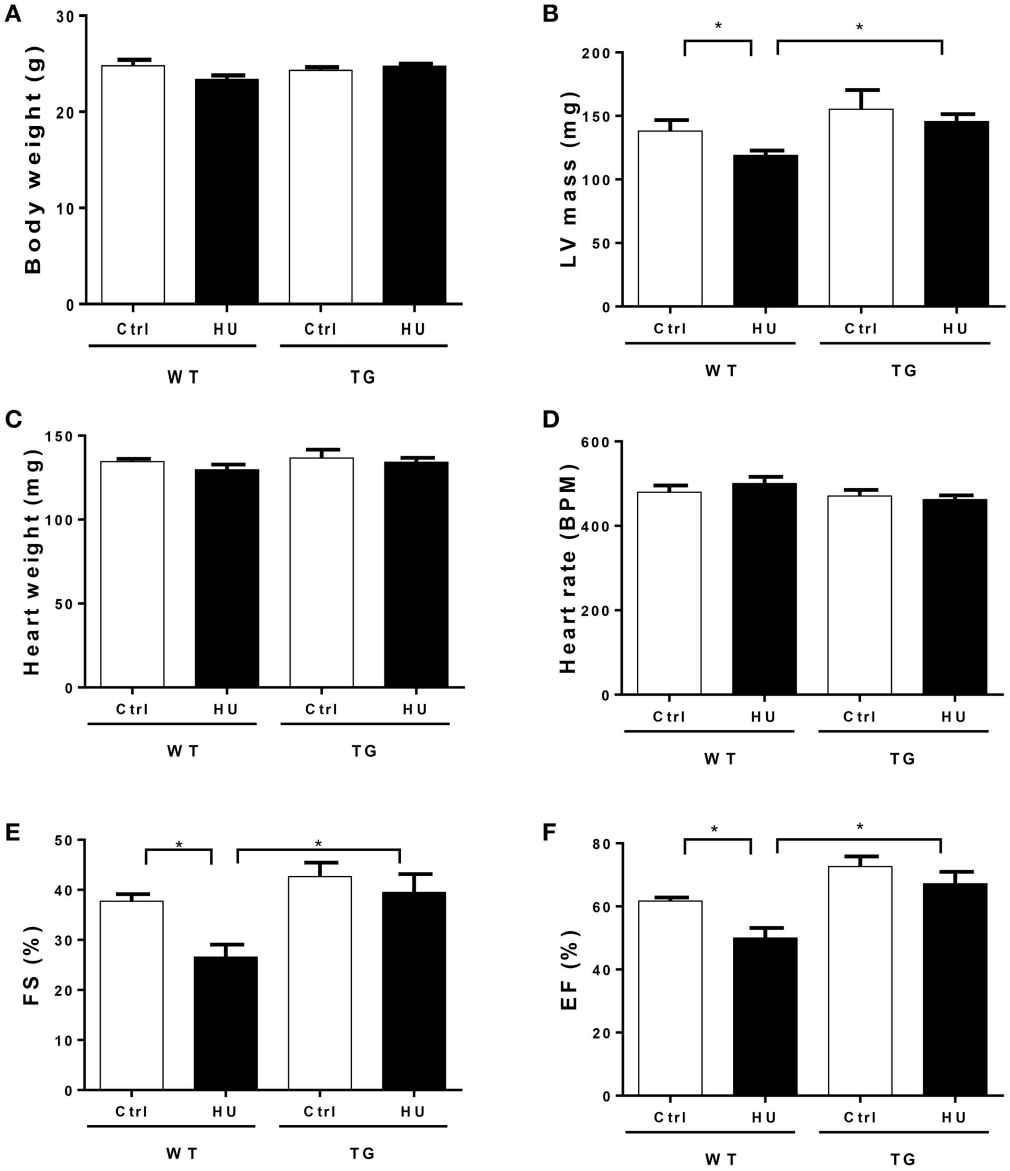
Myocardial CKIP-1 overexpression protects from simulated microgravity induced-the decline of cardiac function. Bodyweight **(A)**, left ventricular mass **(B)**, heart weight **(C)**, and heart rate **(D)** of wild type (WT) and CKIP-1 transgenic (TG) mice after 28 days hindlimb unloading. **(E,F)** Echocardiographic assessment of fractional shortening (FS) and ejection factor (EF) of WT and CKIP-1 transgenic mice after 28 days hindlimb unloading. Ctrl, control group; HU, mice following 28 days of hindlimb unloading; WT, wild type mice, TG, cardiac specific CKIP-1 transgenic mice. Values are means ± SEM, *n* = 6, ^*^P < 0.05.

To further investigate the potential role of CKIP-1 as a suppressor of cardiac remodeling induced by simulated microgravity, we utilized the transgenic (TG) mice with CKIP-1-specific overexpression in cardiomyocytes by using α-MHC promoter. Wildtype (WT) and CKIP-1 TG littermates at 2 months of age were subjected to hindlimb unloading by tail suspension for 28 days. The relevant control groups were treated equally, with the exception of tail suspension. Body weight, heart weight and left ventricular mass were recorded (Figures 2A–C), heart rate and cardiac function were calculated by echocardiography (Figures 2D–F). All echocardiographic measurements were made while the heart rates of mice were maintained at 400–500 beats per minute (Figure 2D). The two way ANOVA was carried out on left ventricular mass, left ventricular fractional shortening (FS), and left ventricular ejection fraction (EF) by condition (control / hindlimb unloading) and genotype (WT/TG). There were all statistically significant interactions between the effects of condition and genotype on left ventricular mass, EF and FS. Compared with the control group, left ventricular mass of WT mice after hindlimb unloading were significantly reduced, meantime left ventricular FS and EF decreased significantly in WT after hindlimb unloading. In comparison with WT mice, CKIP-1 TG mice exhibited a decreased response to hindlimb unload. The changes in left ventricular mass, EF and FS of TG mice after hindlimb were not apparent, and the values were higher than WT-HU group. These results indicated that myocardial CKIP-1 overexpression protected from simulated microgravity induced-decline of cardiac function and loss of left ventricular mass.

### Transthoracic echocardiography assessing the left ventricular structure of WT and TG mice after 28 days of hindlimb unloading

**Figure 3:**
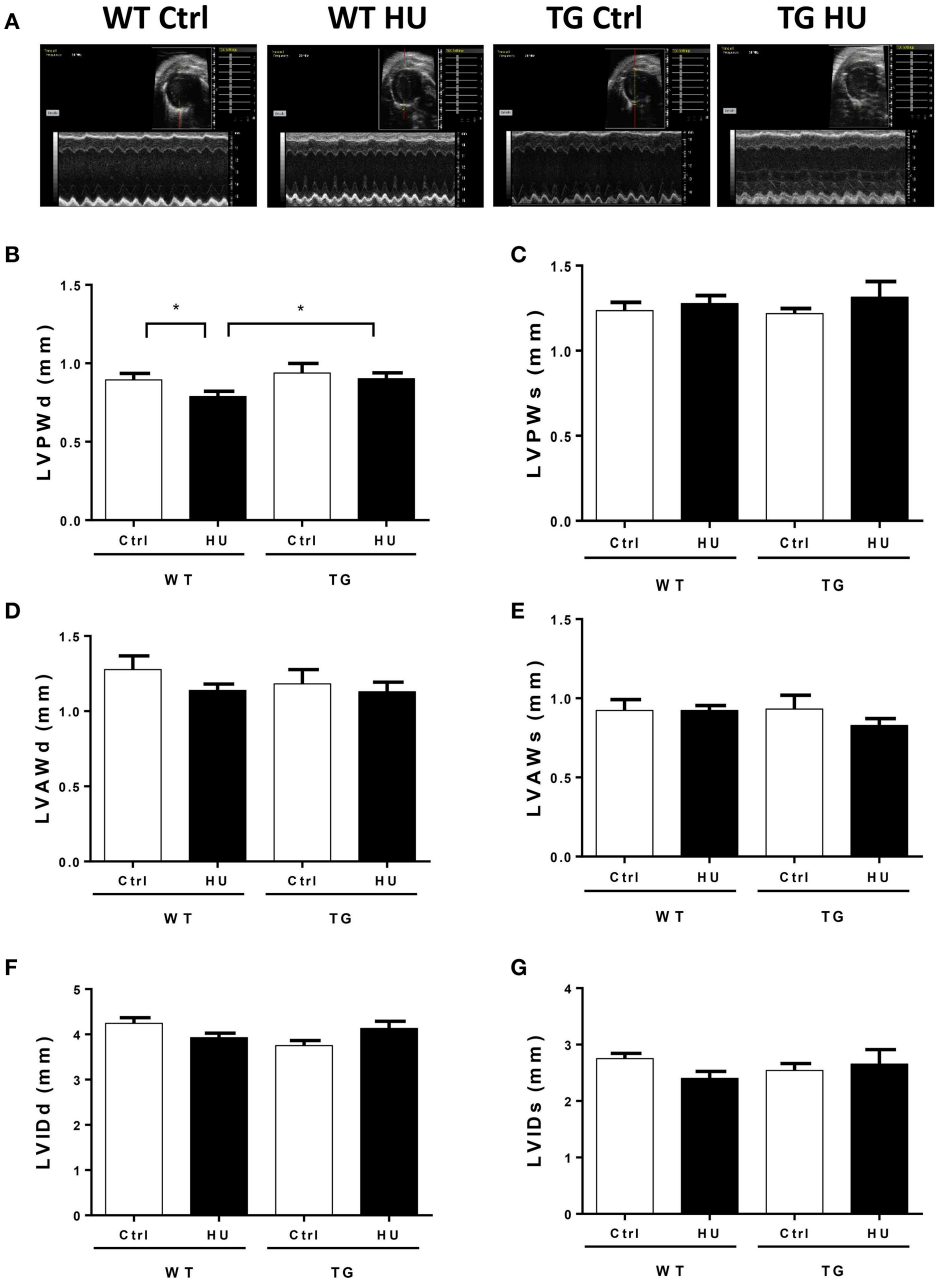
Transthoracic echocardiography evaluating the left ventricular structure of WT and TG mice following hindlimb unloading. **(A)** Representative M-model recordings of echocardiography. **(B–G)** Quantitative analysis of the diastolic and systolic left ventricular posterior wall thickness (LVPWd and LVPWs), LV anterior wal thickness (LVAWd and LVAWs), and LV internal diameter (LVIDd and LVIDs) from WT and TG mice by echocardiography following hindlimb unloading. Values are means ± SEM, *n* = 6, ^*^P < 0.05.

To validate the influence of CKIP-1 TG in the heart structure after hindlimb unloading, we performed transthoracic echocardiography to determine the left ventricular structure of WT and TG mice following hindlimb unloading (Figure 3A). A two way ANOVA was carried out on the end-diastolic left ventricular posterior wall thickness (LVPWd) by condition and genotype. There was a statistically significant interaction between the effects of condition and genotype on LVPWd. Compared with control, LVPWd of WT mice was decreased following hindlmb unloading, however LVPWd of TG mice after hindlimb unloading did not change, and the value was higher than that in WT mice after hindlimb unloading (Figure 3B). Meantime, the end-systolic left ventricular posterior wall thickness (LVPWs) (Figure 3C), the end- diastolic anterior wall thickness (LVAWd) (Figure 3D), the end-systolic anterior wall thickness (LVAWs) (Figure 3E), the end- diastolic LV internal diameter (LVIDs) (Figure 3F), and the end-systolic LV internal diameter (LVIDd) (Figure 3G) did not change in the WT and TG mice after hindlimb unloading. These data indicated that CKIP-1 TG protected from the shortening of LVPWd induced by hindlimb unloading.

### Myocardial CKIP-1 overexpression protects from simulated microgravity induced-cardiac atrophy

**Figure 4:**
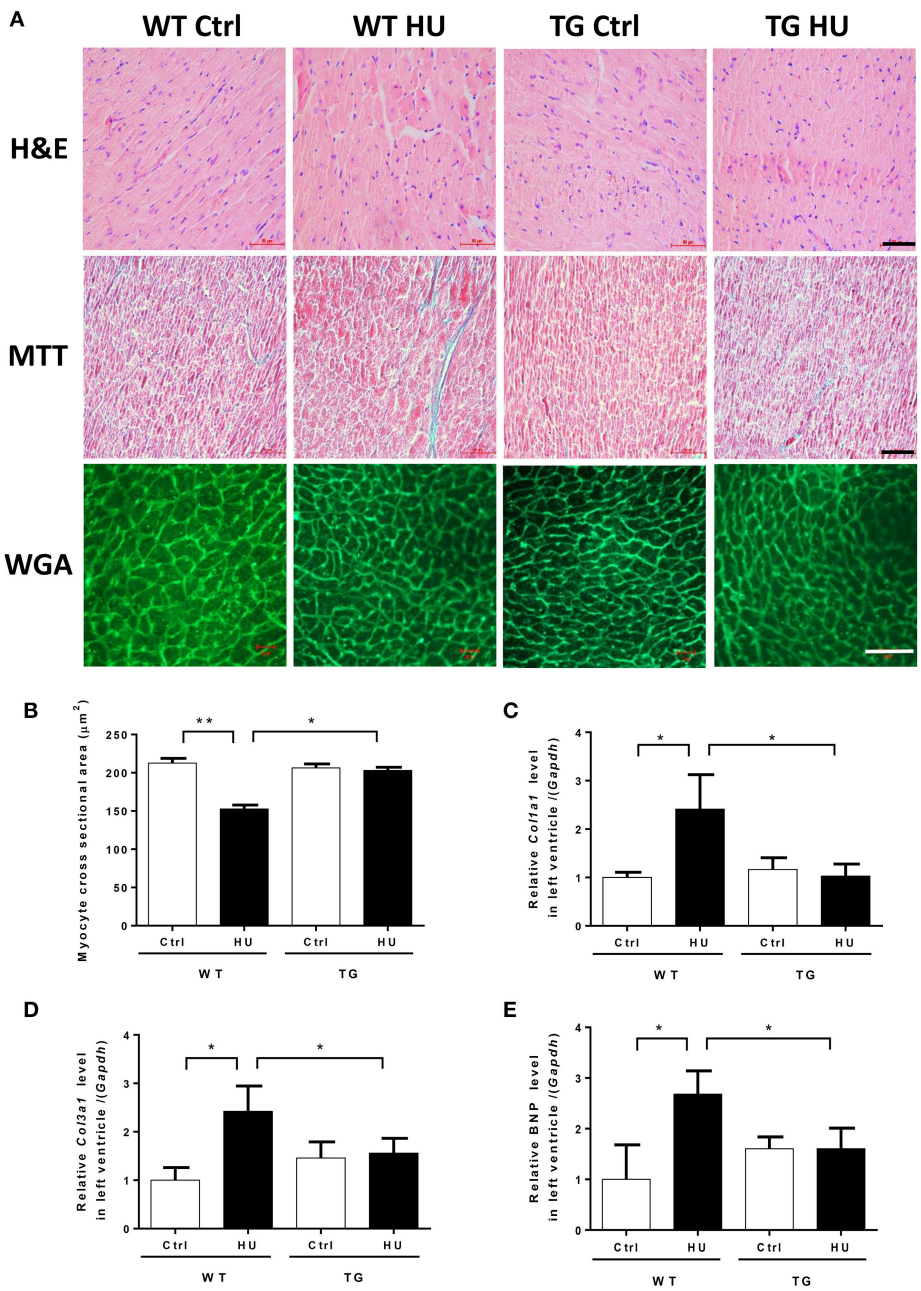
Myocardial CKIP-1 overexpression protects from simulated microgravity induced-cardiac atrophy. **(A)** H&E-stained sections of hearts from WT and CKIP-1 TG mice after 28 days of hindlimb unloading. Sections of hearts are stained with Masson trichrome (MTT) to detect fibrosis (blue). Wheat germ agglutinin (WGA) staining is used to demarcate cell boundaries. Scale bars: 50 μm. **(B)** The cardiomyocyte crosssectional area was measured from 8-μm-thick heart sections that had been stained with WGA by using ImageJ software (NIH). Only myocytes that were round were included in the analysis. The studies and analysis were performed blinded as to experimental. Data represent the means ± SEM (*n* = 6), ^*^P < 0.05, ^**^P < 0.01. **(C–E)** The mRNA levels of Col1a1, Col3a1, and BNP were analyzed by Q-PCR from WT and CKIP-1 TG mice after 28 days of hindlimb unloading. The relative abundance of transcripts were quantified and normalized to GAPDH. Data represent the means ± SEM (*n* = 6), ^*^P < 0.05.

To address the effect of myocardial CKIP-1 overexpression on cardiac atrophy induced by hindlimb unloading, hearts from WT and TG mice were assessed for changes in morphology and cardiac remodeling genes expression. As shown in Figure 4A, in hematoxylin and eosin-stained (H&E) sections, gross evidence of edema was easily observed by separation of the myofibers in the WT mice after hindlimb, however TG mice after hindlimb had no obvious change. Masson trichrome staining (MTT) showed a deeper staining of collagen in the heart of mice after hindlimb unloading, and CKIP-1 TG could inhibit this change. A two way ANOVA was carried out on myocyte cross sectional area by condition and genotype. There was a statistically significant interaction between the effects of condition and genotype on myocyte cross sectional area. Histological analysis demonstrated decreases in the size of individual cardiomyocytes of WT mice after hindlimb unloading, however, the cardiomyocytes of TG mice after hindlimb unloading had no obvious changes compared with control mice, and higher than WT HU group (Figure 4B). The two way ANOVA analysis reports showed there were statistically significant interactions between the effects of condition and genotype on the levels of Col1a1, Col3a1, and BNP mRNAs. The results showed that transcripts for the pathological cardiac remodeling genes-Col1a1, Col3a1, and BNP were significantly increased in the hearts of WT mice after hindlimb unloading, compared with WT control group. And myocardial CKIP-1 overexpression protected from the increases of cardiac remodeling genes expression induced by simulated microgravity (Figures 4C–E).

### Myocardial CKIP-1 overexpression inhibits the changes of signaling pathway in mice heart induced by simulated microgravity

**Figure 5:**
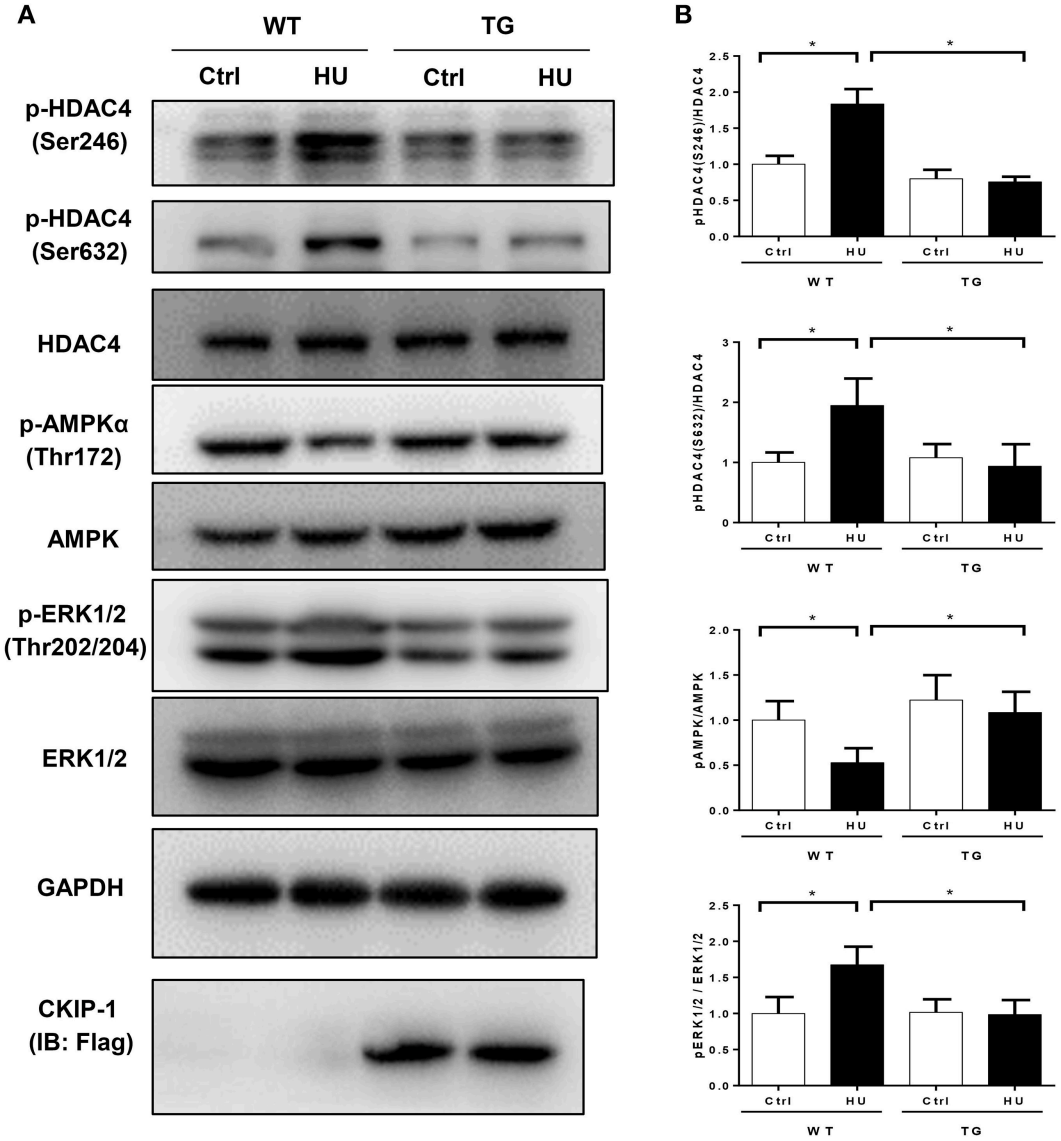
Myocardial CKIP-1 overexpression inhibits the phosphorylation of signal factors in mice heart induced by simulated microgravity. **(A)** Representative Western blots for HDAC4 and phosphorylation at Ser246, AMPKα and phosphorylation at (Thr172), and ERK1/2 and phosphorylation at (Thr202/Tyr204) in hearts from WT and CKIP-1 TG mice after 28 days of hindlimb unloading. Gapdh levels served as a loading control. **(B)** Quantification of phosphorylation levels normalized to total protein levels of heart, Values are means ± SEM (*n* = 6), ^*^P < 0.05.

To gain more insights into the effect of CKIP-1 overexpression on the signaling pathways involved in the cardiac remodeling induced by simulated microgravity, we examined the phosphorylation levels of AMPK, ERK1/2, and HDAC4 in heart tissues of WT and TG mice after hindlimb unloading (Figure 5A). The two way ANOVA analysis reports showed there were statistically significant interactions between the effects of condition and genotype on the phosphorylation levels of HDAC4 phosphorylation at Ser246 and Ser632, Erk1/2 phosphorylation at Thr202/Tyr204 and AMPK phosphorylation at Thr172. As shown in Figure 5B, quantifications of phosphorylation levels normalized to total proteins in the heart revealed HDAC4 phosphorylation at Ser246 and Ser632 and Erk1/2 phosphorylation at Thr202/Tyr204 were increased in WT mice after hindlimb unloading, but it had no obvious changes in TG mice after hindlimb unloading. The phosphorylation level of AMPK at Thr172 was decreased in WT HU group compared with WT control group, but CKIP-1 TG inhibited this change. These results indicated that myocardial CKIP-1 overexpression inhibited the changes in phosphorylation levels of signal factors in mice heart induced by simulated microgravity.

## Discussion

Here, we identified CKIP-1 as a novel regulator of simulated microgravity–induced cardiac remodeling. CKIP-1 mRNA and protein levels were significantly downregulated in the hearts of mice after 28 days of hindlimb unloading and rhesus monkeys after 42 days of head-down bed rest. Myocardial CKIP-1 overexpression protected from simulated microgravity induced-decline of cardiac function and loss of left ventricular mass. Histological analysis demonstrated CKIP-1 TG inhibited the decrease in the size of individual cardiomyocytes of mice after hindlimb unloading. Moreover, the pathological cardiac remodeling signals, such as ERK1/2 and HDAC4, were activated, and physiological cardiac remodeling signals, such as AMPK, were inactivated in heart of WT mice after hindlimb unloading, however CKIP-1 TG mice displayed a different trend. Myocardial CKIP-1 overexpression inhibited the changes of phosphorylation levels of signal factors in mice heart induced by simulated microgravity. So, CKIP-1 manifests important functional significance during cardiac stress response resulting from space flight.

The cardiac muscle is well regulated in response to changes in loading conditions (Hill and Olson, 2008). With prolonged pressure overload, the heart undergoes pathologic hypertrophic remodeling, resulting in dilatation of the failing heart (Hill and Olson, 2008; Ling et al., 2017). Cardiac atrophy was a complication for prolonged microgravity during space flight and mechanical unloading with a ventricular assist device (Levine et al., 1997; Hill and Olson, 2008; Westby et al., 2016). When exposed to 10 days of spaceflight, left ventricular mass decreased by 12 ± 6.9% (Hill and Olson, 2008). After 6 weeks of bed rest, the left ventricular mass decreased by 8.0 ± 2.2%. Thus, cardiac atrophy can be induced by short-term spaceflight or prolonged bed rest (Perhonen et al., 2001). Hindlimb unloading is widely utilized to study the effects of microgravity in mice or rats (Respress et al., 2014; Zhong G. et al., 2016), and head-down tilt bed rest model for non-human primate- rhesus monkeys or human volunteers is also a classical ground-based model of microgravity (Wang et al., 2012; Chen et al., 2016; Ling et al., 2017). The hindlimb of mice are lifted by tail suspension to generate 30-degree head-down tilt for 28 days, and rhesus monkeys were maintained 10-degree head-down tilt position for 42 days. The tilt and unloading of the hindquarters leads to a shift in body fluids toward the head, cardiac remodeling and other physiological changes, which is similar to what is founded in humans during space flight (Zhong G. et al., 2016). In a rodent hindlimb unloading model, the decrease of left ventricular mass occurred within 21 days (Bigard et al., 1994). We previously showed simulated microgravity could induce remodeling of the left and right ventricle of mice (Zhong G. et al., 2016). Cardiac atrophy although is associated with distinct phenotypes (atrophy vs. hypertrophy), generate strikingly similar changes-upregulation of cardiac remodeling marker genes and decline of cardiac function (Depre et al., 1998). As with other form of cardiac remodeling, little is known about the specific mechanism governing the microgravity-induced cardiac atrophy. It is critical to understand the mechanisms regulating cardiac remodeling during microgravity-induced myocardial atrophy in addition to those at play during hypertrophy. Most of the researches suggested that hindlimb unloading can simulate the effect of microgravity which caused a systemic stress, such as fluid shift, and lead to cardiac remodeling and the decline of cardiac function (Watenpaugh, 2002; Zhong G. et al., 2016; Zhong G. H. et al., 2016). In this study, LVPWd of WT mice was decreased following hindlmb unloading, however LVPWd of TG mice after hindlimb unloading did not change, and the value was higher than that in WT mice after hindlimb unloading, TG mice also inhibit the decline of cardiac function induced by simulated microgravity.

Well-characterized signaling molecules that regulate cardiac remodeling induced by pressure overload include HDAC4 (Ling et al., 2012), AMPK (Kovacic et al., 2003), and ERK1/2 (Liu and Hofmann, 2004). HDAC4 shuttles between the cytoplasm and the nucleus in a phosphorylation-dependent manner (Kurdi and Booz, 2011). The nuclear export of HDAC4 can inhibit the transcriptional activity of myocyte enhancer factor-1 (MEF2) which is a master positive regulator of cardiac hypertrophy (Zhang et al., 2002; Kong et al., 2006; Ago et al., 2008). AMPK can regulate cardiac homeostasis, and is a key factor of physiological cardiac remodeling (Schisler et al., 2013; Daskalopoulos et al., 2016). ERK1/2 are members of mitogen-activated protein kinase (MAPK) family, and their activation can regulate pathological cardiac remodeling and heart failure (Ogata et al., 2014; Gu et al., 2016). We previously showed that the pathological cardiac remodeling signals, such as HDAC4 and ERK1/2, were activated, and physiological cardiac remodeling signals, such as AMPK, were inactivated in heart of WT mice after hindlimb unloading (Zhong G. et al., 2016). In the present study, we demonstrated that following 45 days of bed rest, pathological cardiac remodeling signals-ERK1/2 and HDAC4 were activated, and physiological cardiac remodeling signal-AMPK were inactivated in monkey hearts. And the levels of CKIP-1 expression were significantly decreased in the hearts of mice and rhesus monkeys after simulated microgravity. We previously demonstrated that CKIP-1 was a novel regulator of cardiac hypertrophy induced by pressure overload. CKIP-1 TG mice exhibited a strong reduction of pathological cardiac hypertrophy. CKIP-1 functioned as a suppressor of pressure overload-induced cardiac remodeling by upregulating the dephosphorylation and nucleus retention of HDAC4 (Ling et al., 2012). CKIP-1 can interact with HDAC4 and PP2A, increases the interaction between HDAC4 and PP2A, and enhances the activity of PP2A, thus promoting the dephosphorylation of HDAC4 (Ling et al., 2012). Moreover, PP2A can also interact with ERK1/2 in cardiomyocytes, PP2A decreases H_2_O_2_-induced ERK1/2 activation (Liu and Hofmann, 2004). CKIP-1 may also inhibit the phosphorylation of ERK1/2 via PP2A. The phosphorylation of AMPK can be negatively regulated by Akt in heart (Kovacic et al., 2003), furthermore, CKIP-1 plays a critical role in the regulation of macrophage homeostasis by inhibiting Akt activation (Zhang et al., 2014). We speculate that CKIP-1 can enhance the phosphorylation of AMPK via inhibiting Akt activity. In this study, CKIP-1 TG prevented from simulated microgravity-induced cardiac atrophy, as evidenced by gravimetric, echocardiographic, and cell size analysis. CKIP-1 TG can inhibit the activation of HDAC4 and ERK1/2 and the inactivation of AMPK in heart of mice induced by simulated microgravity.

Taken together, our present work has demonstrated that CKIP-1 was a suppressor of cardiac remodeling induced by simulated microgravity. This study provides evidence of the important role of CKIP-1 in cardiac atrophy. These results suggest a novel strategy for positively affecting cardiac remodeling induced by space flight through the upregulation of CKIP-1 in the heart. Nutritional and/or pharmacological interventions may be exploited to prevent cardiac remodeling induced by microgravity via upregulation of CKIP-1 expression in the heart.

## Author contributions

YL and SL: conceived the study; YL and GZ: performed the experiment with support from YZ and HL; QX: performed the transthoracic echocardiography; WS, JL, HS, and DC: analyzed and interpreted the results; DZ, JS, XJ, CL, and XY: provided intellectual contribution; XW, YZ, ZL, and QL: performed the statistical analysis; SL: wrote the manuscript; YL: revised the manuscript and gave final approval of the submitted manuscript; All authors have reviewed and approved the final manuscript.

### Conflict of interest statement

The authors declare that the research was conducted in the absence of any commercial or financial relationships that could be construed as a potential conflict of interest.
