# Supplementary material for: Myocardial CKIP-1 Overexpression Protects from Simulated Microgravity-Induced Cardiac Remodeling
Source: Front Physiol. 2018 Jan 25;9:40. doi: 10.3389/fphys.2018.00040 (PMC5788970; doi:10.3389/fphys.2018.00040)

Myocardial CKIP-1 Overexpression Protects from Simulated Microgravity-Induced Cardiac Remodeling

**Shukuan Ling[1#]; Yuheng Li[1#]; Guohui Zhong[1#]; Yongjun Zheng[2]; Dingsheng Zhao[1]; Qing Xu[3]; Hongxing Li[4]; Weijia Sun[1]; Jianwei Li[1]; Huiyuan Sun[5]; Dengchao Cao[6]; Jinping Song[1]; Xiaoyan Jin[1]; Caizhi Liu[1]; Xinxin Yuan[6]; Xiaorui Wu[1]; Yinlong Zhao[4]; Zizhong Liu[1]; Qi Li[1]; Yingxian Li[1]*

[1]State Key Lab of Space Medicine Fundamentals and Application, China Astronaut Research and Training Center, Beijing 100094, China.

[2] Medical Administration Division, the 261th Hospital of PLA, Beijing 100094, China.

[3]Core Facility Center, Capital Medical University, Beijing 100069, China.

[4]Key Laboratory of Molecular and Cellular Biology of Ministry of Education, College of Life Science, Hebei Normal University, Shijiazhuang 050024, China.

[5]Xiyuan Hospital, China Academy of Chinese Medical Sciences, Beijing 100091, China.

[6]State Key Laboratory of Agrobiotechnology, College of Life Sciences, China Agricultural University, Beijing 100193, China.

# These authors have contributed equally to this work.

* Address for Correspondence:

Yingxian Li, Ph.D., State Key Lab of Space Medicine Fundamentals and Application, China Astronaut Research and Training Center, No. 26 Beiqing Road, Haidian District, Beijing, 100094, China. Tel: 8610-62895755, Fax: 8610-62895755, E-mail: yingxianli@aliyun.com

Supplementary Figure 1

The mRNA levels of Col1a1, Col3a1, BNP, β-MHC and α-MHC were analyzed by Q-PCR from the left ventricular of rhesus monkeys following 45 days of head-down bed rest. The relative abundance of transcripts were quantified and normalized to GAPDH. Data represent the means ± SEM (n=3), *P<0.05.

Supplementary Figure 2

Activity of signaling pathways in the left ventricular of rhesus monkeys following 45 days of head-down bed rest. Western blots for HDAC4 and phosphorylation at Ser246, AMPKα and phosphorylation at (Thr172), and ERK1/2 and phosphorylation at (Thr202/Tyr204) in left ventricular of rhesus monkeys with or without bed rest.

Supplementary Figure 1

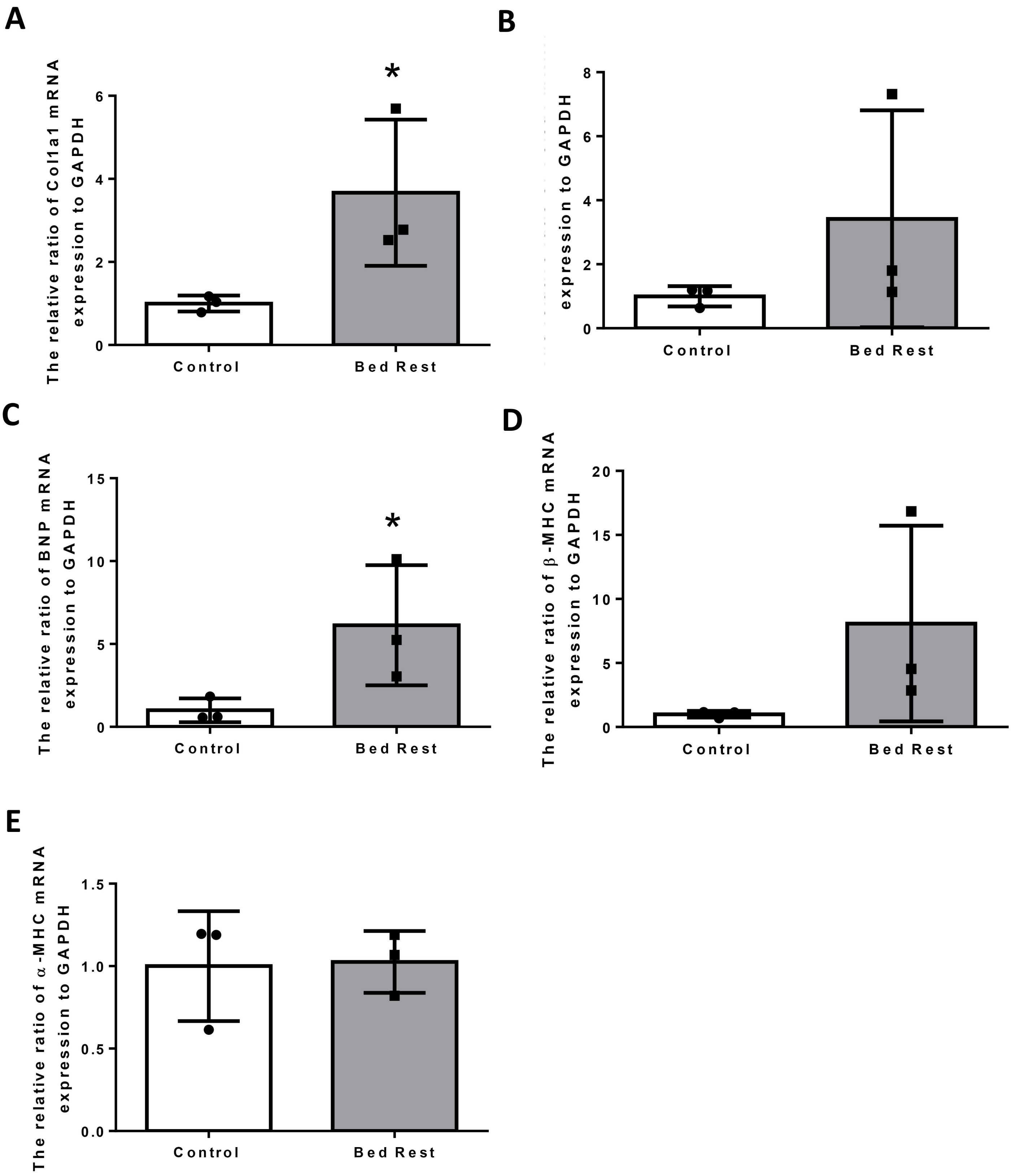

Supplementary Figure 2

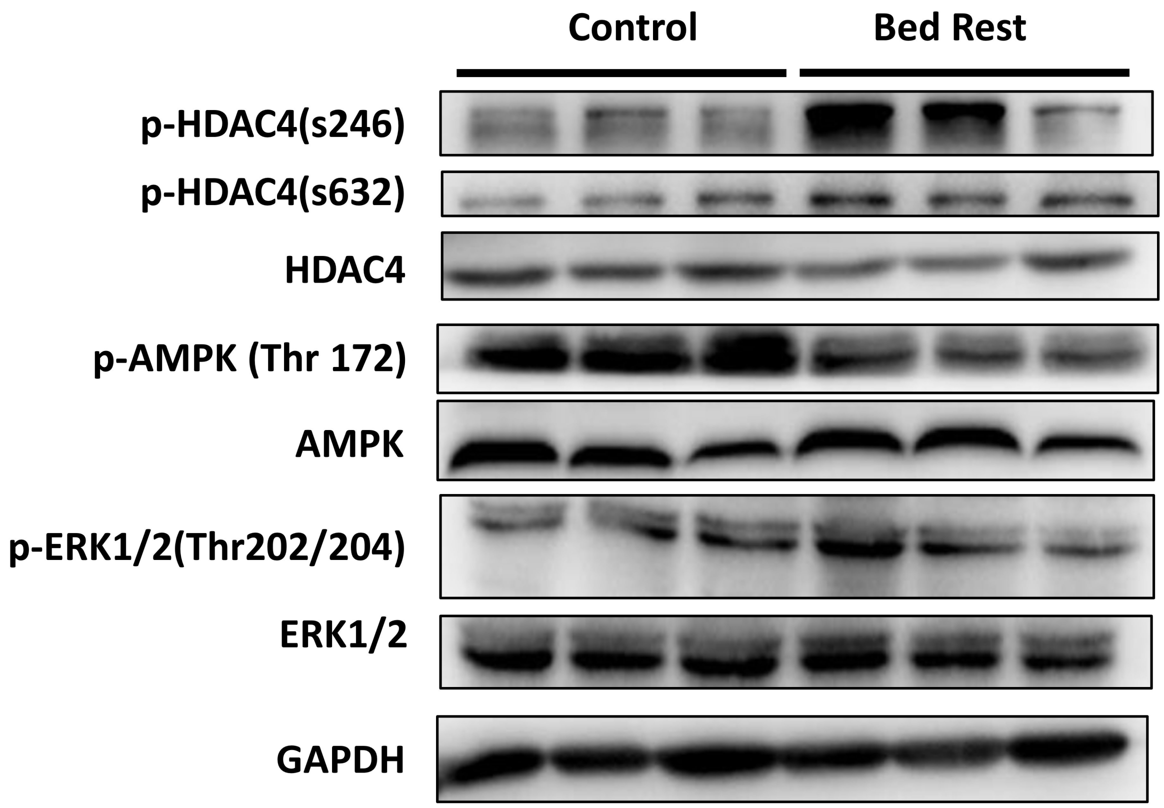

Fig 1B

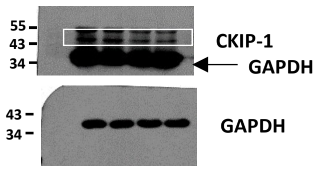

Fig 1D

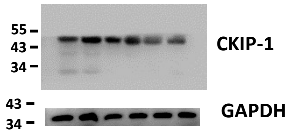

Sup Fig

Fig 5A

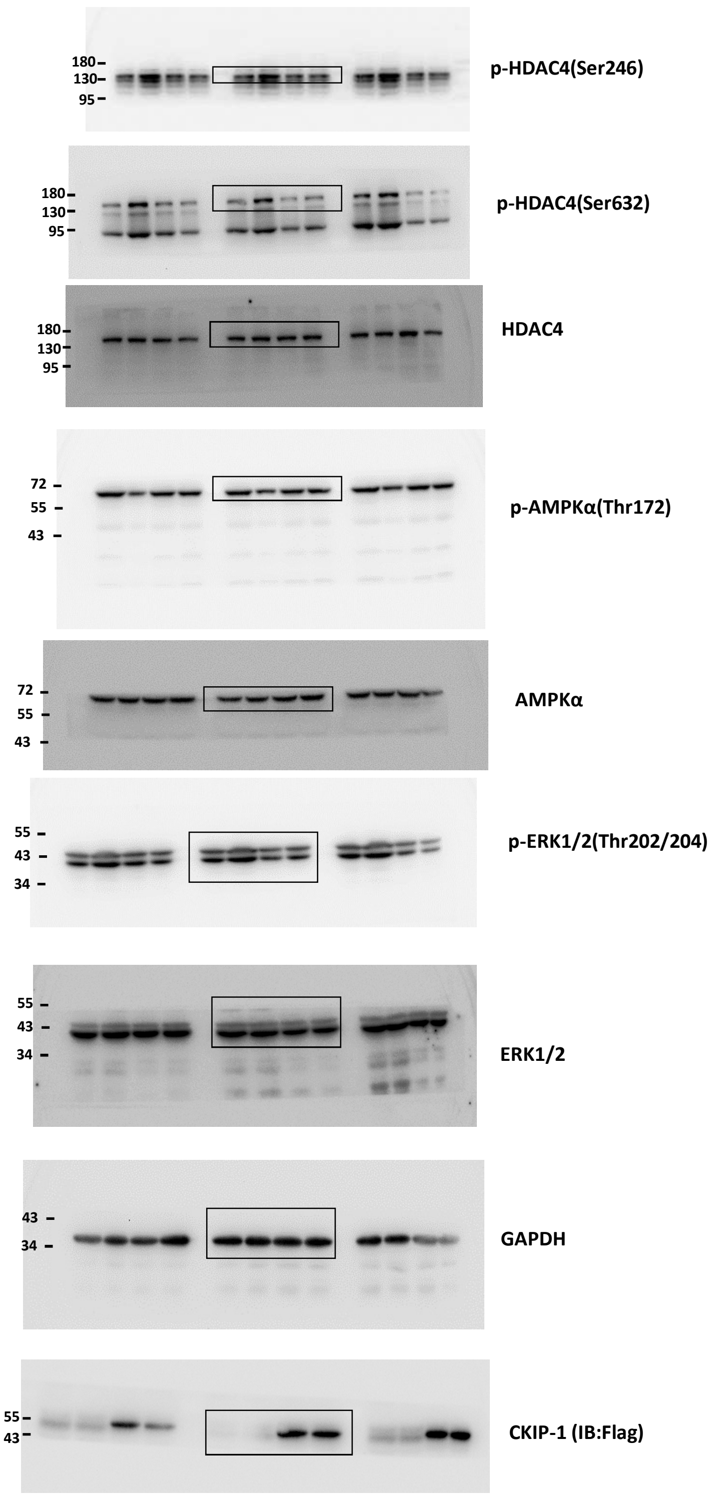

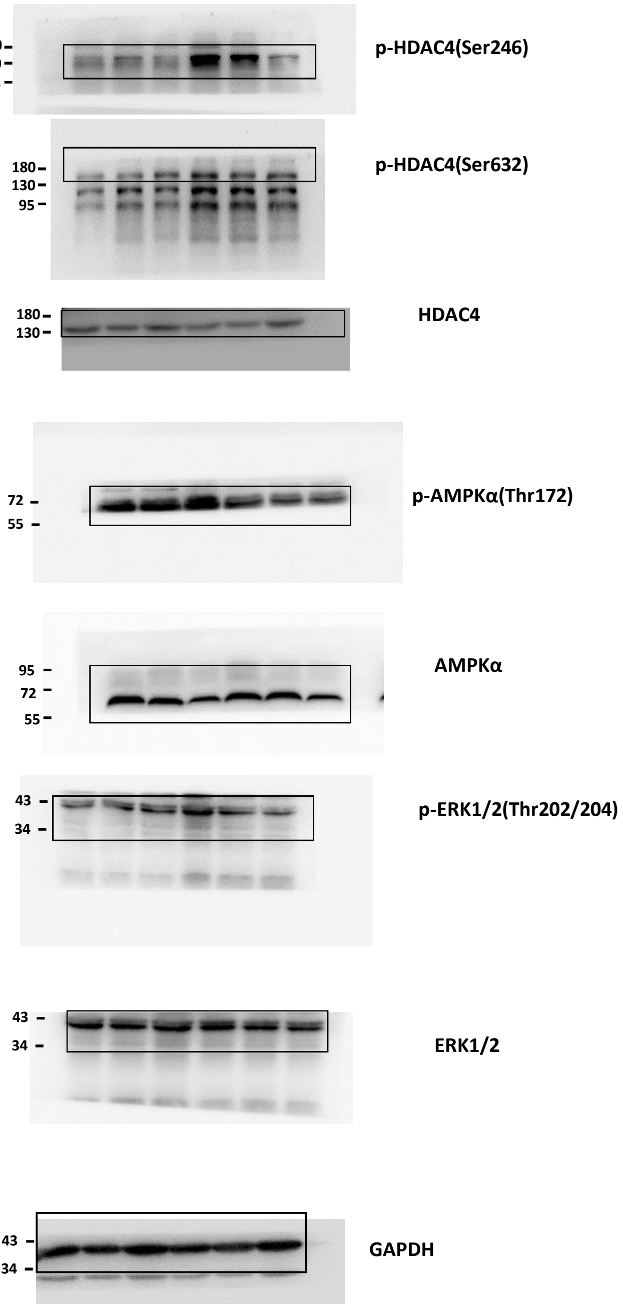

Supplement: Supplementary file 1 [file Presentation1.PDF]
